# Gender differences in left ventricular geometry and determinants of myocardial perfusion reserve in patients with severe aortic stenosis

**DOI:** 10.1186/1532-429X-13-S1-O41

**Published:** 2011-02-02

**Authors:** Christopher D Steadman, Michael Jerosch-Herold, Benjamin Grundy, Suzanne Rafelt, Leong L Ng, Iain B Squire, Nilesh J Samani, Gerry P McCann

**Affiliations:** 1 Department of Cardiovascular Sciences, University of Leicester, Leicester, UK; 2 Department of Radiology, Brigham & Women's Hospital and Harvard Medical School, Boston, MA, USA; 3 NIHR Leicester Cardiovascular Biomedical Research Unit, Leicester, UK

## Objectives

The aim of this analysis was to look at the impact of gender on LV geometry and the predictors of myocardial perfusion reserve (MPR) in severe aortic stenosis (AS).

## Background

It is well recognised that cardiac size is different between the two genders, even when corrected for body size. In AS echocardiographic studies suggest women have higher relative wall thickness and better preserved left ventricular (LV) ejection fraction (EF). There are little cardiac magnetic resonance (CMR) data on gender differences in severe AS, in particular MPR.

## Methods

Forty-one patients with isolated severe AS without obstructive coronary artery disease underwent adenosine stress perfusion CMR in a 1.5T scanner (Siemens Avanto); MPR was calculated from absolute myocardial blood flow during adenosine hyperaemia and rest determined by model-independent deconvolution of signal intensity curves with an arterial input function. Transthoracic echocardiography was used to assess AS severity, tissue Doppler derived diastolic function, LVRPP (LV rate pressure product=[systolic blood pressure(SBP) + peak aortic valve gradient]*heart rate) an estimate of myocardial work, and diastolic perfusion time (DPT=[R-R interval - LV ejection time]*heart rate).

## Results

Females were older with significantly lower body surface area (BSA) and higher SBP. Despite equivalent AS severity females had significantly lower LV mass index (LVMI), LV volumes and wall thickness (relative and absolute), less late gadolinium enhancement (LGE) and higher EF. Resting and hyperaemic myocardial blood flow (MBF) were higher in females although MPR remained the same. Results are summarised in Tables 1 and 2. Variables with the strongest correlation with MPR in females were mean pressure gradient (PG), LGE, LVRPP, and DPT in contrast to LV mass, relative wall thickness and septal E/E’ in males, Table 3.

**Table 1:** Demographics and Echocardiographic Data

Variable	Whole group (n=41)	Male (n=31)	Female (n=10)	Male vs. Female p-value
Age	66±8	64±8	72±8	0.018*
BSA(m2)	1.92±0.21	1.98±0.18	1.71±0.14	0.001*
SBP(mmHg)	130±19	126±15	144±23	0.008*
Peak aortic velocity (AV)(m/s)	4.42±0.58	4.45±0.60	4.33±0.52	0.564
Mean PG(mmHg)	47.9±14.3	48.6±15.0	45.9±12.2	0.604
Aortic valve area index (AVAI) (cm2/m2)	0.46±0.13	0.44±0.10	0.50±0.20	0.219
LVRPP(mmHg.bpm.10-4)	1.51±0.28	1.47±0.29	1.64±0.19	0.101
Resting DPT(s/min)	37.8±4.0	38.4±3.7	35.8±4.2	0.074
Septal E/E’	14.1 [12.2-18.6]	12.9 [10.9-18.4]	16.2 [15.2-18.9]	0.052

**Table 2:** CMR Data

Variable	Whole group (n=41)	Male (n=31)	Female(n=10)	Male vs. Female p-value
LVMI(g/m2)	68.9±17.9	74.1±16.3	52.8±12.5	0.001*
LV end-diastolic volume index (LVEDVI) (mL/m2)	96.4±15.2	99.8±13.5	85.8±16.0	0.009*
LVM/LVEDV(g/mL)	0.72±0.15	0.74±0.14	0.62±0.13	0.025*
LVEF(%)	56.4±6.5	54.9±6.8	60.5±5.0	0.022*
Maximum wall thickness(mm)	13.2±2.5	13.8±2.4	11.1±1.4	0.002*
Resting MBF(mL/min/g)	0.90±0.20	0.84±0.15	1.06±0.24	0.001*
Hyperaemic MBF(mL/min/g)	1.77±0.47	1.64±0.38	2.15±0.53	0.002*
MPR	2.03±0.55	2.01±0.53	2.09±0.63	0.674
LGE present	23 (56%)	21 (68%)	2 (20%)	0.012*

**Table 3:** Correlations with MPR

Variable	Male Beta	Male p-value	Female Beta	Female p-value
Peak AV	-0.349	0.059	-0.361	0.324
Mean PG	-0.277	0.136	-0.524	0.158
AVAI	0.258	0.165	0.098	0.807
LVMI	-0.495	0.005*	-0.348	0.340
LVM/LVEDV	-0.477	0.008*	-0.105	0.842
LGE	-0.332	0.071	-0.629	0.071
LVRPP	-0.103	0.595	0.768	0.093
DPT	-0.340	0.078	-0.521	0.136
Septal E/E’	-0.527	0.004*	-0.227	0.545

## Conclusions

These findings confirm the gender differences in LV geometry in response to pressure overload. The factors contributing to microvascular dysfunction also appear to be different. In males increased LV mass and relative wall thickness with associated diastolic dysfunction appear to be important determinants of microvascular dysfunction, in contrast to females where pressure gradients and diastolic perfusion time play a larger role. Paradoxically LVRPP was positively correlated with MPR in females. These differences may have implications for the treatment of microvascular dysfunction in females compared with males.

